# Gene Expression Profile of Placenta and Adipose Tissue in Women with Gestational Diabetes Mellitus

**DOI:** 10.3390/ijms26199595

**Published:** 2025-10-01

**Authors:** Renata Saucedo, Erika Magallón-Gayón, Rocio Alejandra Chavez-Santoscoy, Mary Flor Díaz-Velázquez, Aldo Ferreira-Hermosillo, Diana Ojeda-López, Wendy Porras-Marcial, Debbie López-Sánchez, Jorge Valencia-Ortega

**Affiliations:** 1 Unidad de Investigación Médica en Enfermedades Endocrinas, Hospital de Especialidades, Centro Médico Nacional Siglo XXI, Instituto Mexicano del Seguro Social, Mexico City 06720, Mexico; renata.saucedo@imss.gob.mx (R.S.); aldo.nagisa@gmail.com (A.F.-H.); 2 Escuela de Ingeniería y Ciencias, Campus Monterrey, Tecnológico de Monterrey, Ave. Eugenio Garza Sada 2501 Sur, Monterrey 64700, Mexico; erika.magallon@tec.mx (E.M.-G.); chavez.santoscoy@tec.mx (R.A.C.-S.); 3 Hospital de Gineco Obstetricia 3, Centro Médico Nacional La Raza, Instituto Mexicano del Seguro Social, Mexico City 02990, Mexico; mary.diaz@imss.gob.mx (M.F.D.-V.); dianaojeda95@gmail.com (D.O.-L.); michell.vip@hotmail.com (W.P.-M.); 4 Sección de Estudios de Posgrado, Escuela Superior de Medicina, Instituto Politécnico Nacional, Mexico City 11340, Mexico; debbiearleth21@gmail.com; 5 Laboratorio de Investigación en Biología Molecular, Hospital Infantil de México “Federico Gómez,” Mexico City 06720, Mexico

**Keywords:** gestational diabetes mellitus, placenta, visceral adipose tissue, RNA-seq signatures, transcriptome

## Abstract

Placenta and visceral adipose tissue (VAT) are implicated in the development of gestational diabetes mellitus (GDM). In the present study, we examined the whole-transcriptomic profile of both tissues in GDM women to elucidate the molecular basis of GDM pathogenesis. The whole-transcriptome profile was analyzed in placenta and VAT from at-term patients with GDM and controls using RNA-seq. qPCR was used to validate several differentially expressed genes (DEGs). A total of 179 DEGs were observed in the placenta and 4 in VAT, including both up- and downregulated genes. The expression of the selected mRNAs for validation was consistent with the sequencing results. An analysis of the placental upregulated DEGs in the GDM women showed enrichment in functions including the G-protein-coupled receptor signaling pathway, organophosphate biosynthetic process, and phospholipid metabolic process, while the downregulated DEGs were enriched in cell motility and the cell migration process. The target pathways of DEGs in VAT are related to cancer and to the activation of the complement cascade. Molecular pathways involved in G-protein-coupled receptor signaling, the organophosphate biosynthetic process, the phospholipid metabolic process, and cell motility and cell migration are altered in the placentas of GDM women. Moreover, a disordered complement cascade might take place in the VAT of GDM women.

## 1. Introduction

Gestational diabetes mellitus (GDM), defined as diabetes diagnosed in the second or third trimester of pregnancy that was not clearly overt diabetes prior to gestation, is the most common metabolic disorder in pregnancy [1]. The prevalence of this complication is 14% worldwide and has increased in parallel with the rise in obesity and maternal age [2]. Gestational diabetes is correlated with adverse pregnancy outcomes, including preeclampsia and macrosomia, and with long-term maternal and offspring cardio-metabolic risk. In addition to this, GDM is also associated with substantial costs [3].

The onset of GDM occurs in the second trimester due to increased insulin resistance and inadequate β-cell compensation [4]. However, the pathophysiological mechanisms underlying GDM are not yet fully known. It has been documented that the placenta and visceral adipose tissue (VAT) are implicated in this disorder [5,6,7]. Several placental-derived hormones have been demonstrated to interfere with insulin signaling, including human placental lactogen, human placental growth hormone, prolactin, estrogen, progesterone, and cytokines secreted by VAT, such as tumor necrosis factor α (TNF-α). These molecules are believed to play important roles in the development of insulin resistance in patients with GDM [4]. However, to date, there have been few studies addressing the molecular mechanisms to clarify the pathophysiology of GDM. Particularly, transcriptional signatures have been investigated both in the placenta and VAT. The transcriptome profile in the placenta of women with GDM has shown that genes involved in cellular growth, proliferation and migration, metabolic pathways, and immune and type 1 diabetes mellitus (T1DM) processes are altered [8,9]. On the other hand, gene expression studies in VAT suggest that immune-related genes and the inflammatory response are associated with GDM [7]. However, to the best of our knowledge, the simultaneous identification of the transcriptome profile of the placenta and VAT using high-throughput RNA sequencing (RNA-seq) in GDM women has not, so far, been studied.

In the present study, we examined the whole-transcriptomic profile of the placenta and omental VAT in GDM women to elucidate the molecular basis of GDM pathogenesis.

## 2. Results

### 2.1. Maternal Characteristics

The clinical characteristics of the NGT and GDM women are summarized in Table 1. There was no significant difference in the maternal age, pre-gestational weight, pre-pregnancy BMI, maternal BMI at delivery, gestational age at birth, maternal glucose at delivery, newborn weight, or infant sex. Meanwhile, one participant in the NGT group and three women in the GDM group were diagnosed with obesity.

### 2.2. Differential Gene Expression Profiles of NGT and GDM Patients

**Figure 1:**
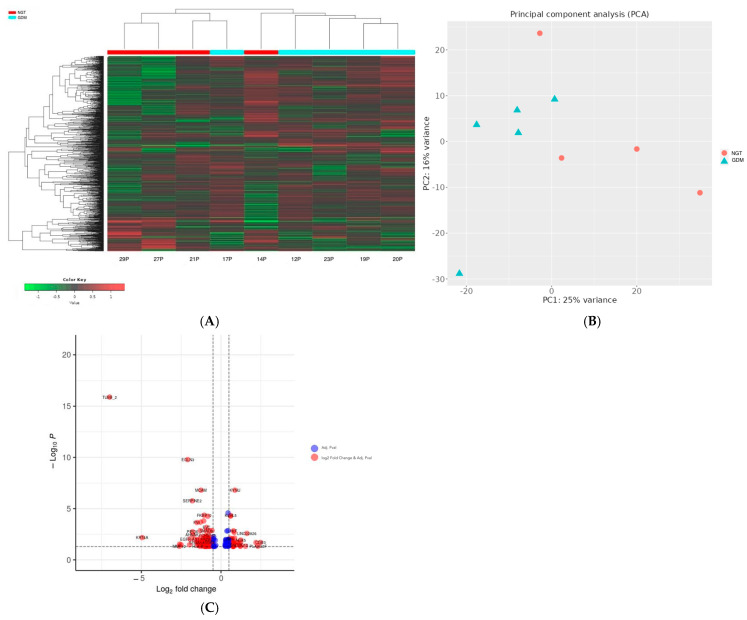
(**A**) Hierarchical clustering heatmap of differentially expressed genes between NGT and GDM samples. The heatmap displays the expression profiles of genes with significant differential expression between NGT (red) and GDM (cyan) groups. Rows represent individual genes, and columns represent samples. Gene expression values are scaled and represented by a color gradient, where red indicates upregulation and green indicates downregulation. (**B**) Principal Component Analysis (PCA). PCA was performed on normalized gene expression data to explore the overall variance among samples. Each point represents a single sample, colored by experimental group: NGT (red circles) and GDM (cyan triangles). (**C**) Volcano plot of differentially expressed genes. Blue dots represent genes that passed the adjusted *p*-value significance threshold, while red dots indicate genes meeting both significance and fold change criteria. Vertical dashed lines represent log_2_ fold change cutoffs (±1), and the horizontal dashed line denotes the *p*-value threshold. Genes significantly upregulated or downregulated in GDM compared to NGT are visibly separated on either side of the plot.

**Figure 2:**
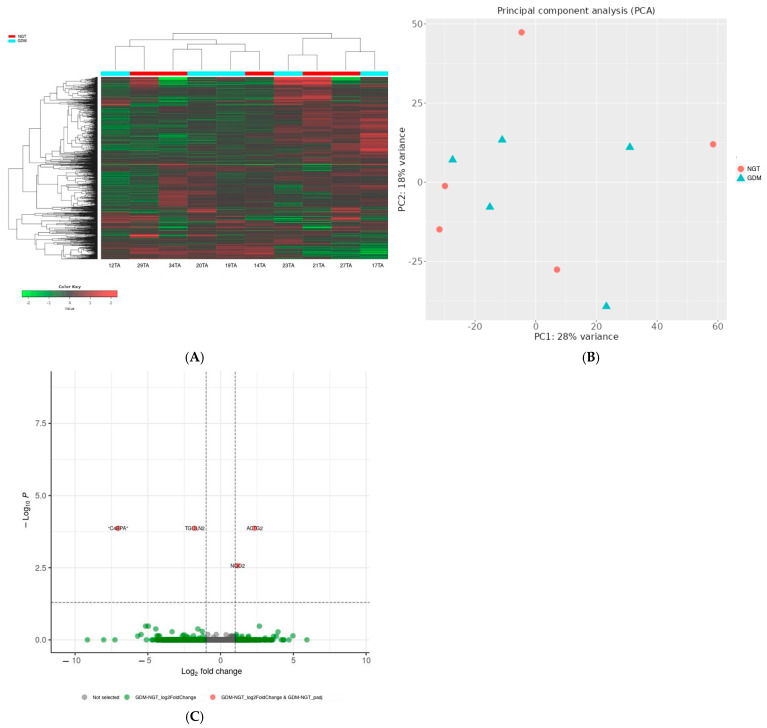
(**A**) Hierarchical clustering heatmap of differentially expressed genes between NGT and GDM samples. The heatmap displays the expression profiles of genes with significant differential expression between NGT (red) and GDM (cyan) groups. Rows represent individual genes, and columns represent samples. Gene expression values are scaled and represented by a color gradient, where red indicates upregulation and green indicates downregulation. (**B**) Principal Component Analysis (PCA). PCA was performed on normalized gene expression data to explore the overall variance among samples. Each point represents a single sample, colored by experimental group: NGT (red circles) and GDM (cyan triangles). (**C**) Volcano plot of differentially expressed genes. Green dots represent genes that passed the fold change (±1), while red dots indicate genes that meet both the fold change and adjusted *p*-value thresholds. Gray dots correspond to genes not passing either criterion. Dashed vertical lines mark log_2_ fold change thresholds (±1), and the horizontal line marks the *p*-value cutoff.

In total, 112 genes were downregulated and 67 genes were upregulated in the placentas of the GDM women compared with those in the NGT group (Figure 1, Supplementary Table S1). Additionally, in the VAT, four DEGs were identified: the *C4BPA* and *TGOLN2* genes were downregulated and *ACTG2* and *NQO2* were upregulated in the GDM women compared with the NGT women (Figure 2).

### 2.3. Gene Set Enrichment Analysis

**Figure 3:**
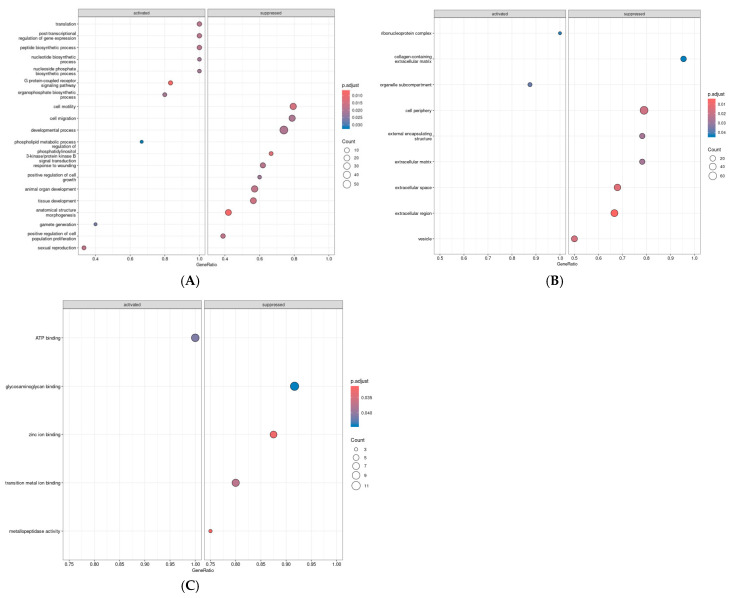
Functional enrichment analysis of differentially expressed genes between GDM and NGT groups. Dot plots display enriched Gene Ontology (GO) terms among differentially expressed genes, categorized into (**A**) biological process (BP), (**B**) cellular component (CC), and (**C**) molecular function (MF). Terms are grouped into activated (upregulated in GDM) and suppressed (downregulated in GDM) categories. The size of each dot represents the number of genes associated with each GO term (Count), the position on the x-axis represents the gene ratio (proportion of genes involved in the term), and color intensity indicates the adjusted *p*-value of enrichment (FDR corrected).

The biological processes activated in the placentas of the GDM women compared to those of the NGT women were the G-protein-coupled receptor signaling pathway, organophoshate biosynthetic process, and phospholipid metabolic process. The biological processes that were suppressed were cell motility and cell migration. Furthermore, in the cellular component analysis, there was an upregulation of the ribonuclein complex and organelle sub-compartment and a downregulation of the collagen-containing extracellular matrix and cell periphery in the GDM group compared to the NGT group. Finally, in terms of molecular function, ATP binding was activated, and glycosaminoglycan binding and zinc ion binding were suppressed in the GDM group compared to the NGT group (Figure 3, Supplementary Table S2). As only four DEGs were identified in the VAT, we did not perform gene set enrichment analysis in this tissue.

**Figure 4:**
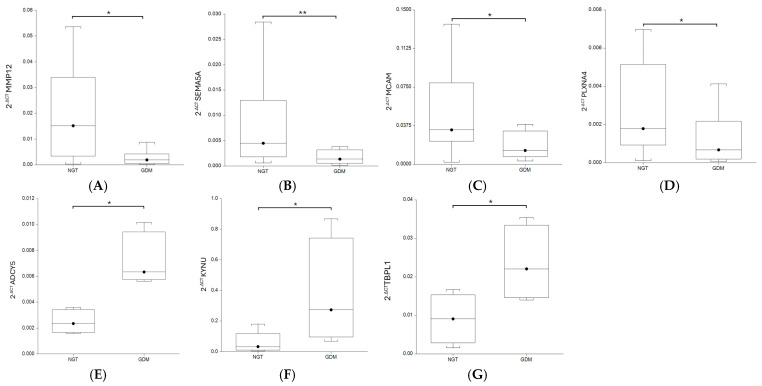
Box and whisker plots of *MMP12*, *SEMA5A*, *MCAM*, *PLXNA4*, *ADCY5*, *KYNU*, and *TBPL1* expression levels in placenta of normal-glucose-tolerance (NGT) women and women with gestational diabetes mellitus (GDM). GDM group showed decreased *MMP12* (**A**), *SEMA5A* (**B**), *MCAM* (**C**), and *PLXNA4* (**D**) expression levels and increased *ADCY5* (**E**), *KYNU* (**F**), and *TBPL1* (**G**) expression levels compared with NGT group. * *p* < 0.05, ** *p* < 0.01.

To validate the expression of dysregulated mRNAs in placenta, we selected 10 downregulated genes associated with cell motility and 4 upregulated genes associated with organophosphate biosynthetic process, and we were able to confirm 7 modifications with either decreased or increased expression. The results indicated that the cell motility gene levels were decreased, and the organophosphate gene levels were increased in the placenta samples of the women with GDM compared with the NGT samples, which were consistent with the transcriptomic sequencing data (Figure 4).

## 3. Discussion

GDM is a common complication during pregnancy that is associated with negative short-term and long-term consequences for both the mother and infant. The mechanism responsible for the development of GDM is currently not very well understood [10]. Increasing transcriptome analysis studies have shown new mediators implicated in the pathogenesis of GDM [11]. In our study, we have found differential gene expression profiles in the placentas and VAT of women with GDM as compared to healthy pregnant patients. However, we noticed greater differences in the placentas than in the VAT. Our findings suggest a major fundamental role of the placenta in GDM. Increased placental size and weight has been reported in GDM patients, accompanied by higher intimate glycogen deposits, increased number of syncytial knots, villous edema, and larger syncytial area and volume to favor nutrient uptake than in normal pregnancies [12]. In the present study, we identified 179 DEGs in the placentas, with most being downregulated in the GDM group. According to the enrichment analysis of the DEGs, among the genes downregulated in the placentas of the GDM women, we identified genes more specifically involved in cell motility and cell migration, suggesting low migration of trophoblasts in GDM. The placenta is a heterogeneous organ; it contains trophoblasts (cytotrophoblast cells, villous cytotrophoblasts, syncytiotrophoblasts, and extravillous trophoblasts) and vascular and immune cells such as lymphocytes and macrophages [13]. Recently, Yang et al. [14], using single-cell RNA sequencing of the placenta of GDM women, reported that trophoblasts were the most abundant cell group in the placenta. The migration of trophoblasts is essential for placenta formation. It has been suggested that hyperglycemia inhibits placentation. Tao et al. [15] showed that HTR-8/SVneo trophoblast cell line migration in high-glucose conditions was probably decreased through the placental growth factor/ROS pathway. Also, Heim et al. [16] showed that hyperglycemia limits trophoblast migration by inducing inflammation. Furthermore, Belkacemi et al. [17] showed that trophoblast invasion was reduced by approximately 62% when HTR-8 cells were treated with 10 mM of glucose. The migration of trophoblasts is mediated by certain genes. We demonstrated a downregulation of *MMP12*, *SEMA5A*, *PLXNA4*, and *MCAM* in the placenta of the GDM patients.

*MMP12* is a member of the peptidase M10 family of matrix metalloproteinases (MMPs). Proteins in this family are involved in the breakdown of the extracellular matrix during implantation, placentation, and the invasion of the trophoblast in the endometrium. *MMP12* is known as a macrophage metalloelastase that leads to the degradation of a wide range of extracellular matrix proteins through its ability to activate other MMPs [18]. Dysregulation of placental MMP expression has been linked with pregnancy complications including fetal growth restriction, preeclampsia (PE), and GDM. A previous study using transcriptome sequencing demonstrated an upregulation of *MMP12* in the third-trimester placenta of individuals with GDM compared to the control group [19]. On the other hand, in our study, we demonstrated a downregulation of *MMP12* using transcriptome sequencing and qRT-PCR. The discrepancy between our findings is unclear but might be related to ethnic differences and population differences in pre-pregnancy weight and glycemic control. In line with that, a downregulation of *MMP14* has been demonstrated in the GDM placentas of obese women [20]. In our study, 40% of the participants were diagnosed with obesity.

*SEMA5A* encodes semaphorin 5A, which is a membrane protein containing a semaphorin domain and several thrombospondin type-1 repeats [21]. Members of the semaphorin family are implicated in neural development and in a variety of functions outside the nervous system [22]. In placenta, *SEMA4A* promotes the migration and proliferation of trophoblast cells and inhibits their apoptosis [23]. Additionally, *SEMA3A* exerts a significant influence on the process of decidualization and relates to unexplained spontaneous miscarriage [24]. Furthermore, *SEMA3* causes low trophoblast invasion and several studies have found increased *SEMA3* levels in PE [25]. On the other hand, *SEMA5A* is involved in cell proliferation and migration. This molecule has been implicated in tumorigenesis, it enhances cell proliferation and decreases apoptosis in gastric cancer, increases metastasis and induces angiogenesis in pancreatic cancer, enhances metastatic progression in prostate cancer, and also increases cervical cancer cell invasion [21]. However, the role of *SEMA5A* in GDM is not known. In our study, we have demonstrated that *SEMA5A* expression was lower in the GDM compared to the NGT women.

Plexins are the main receptors of semaphorins. *PLXNA4* is the functional receptor for *SEMA3A*, and the binding of this semaphorin to its receptor plays a role in endometrial decidualization. This signaling is attenuated in unexplained spontaneous miscarriage [24]. Our data showed that *PLXNA4* expression was lower in the GDM group compared with the NGT participants. We can speculate that this could be due in part to the low expression of the *PLXNA4* ligand.

*MCAM*, melanoma cell adhesion molecule, is a cell adhesion molecule belonging to the immunoglobulin supergene family. This molecule is detected mainly in melanoma, vascular endothelial cells, smooth muscle, trophoblasts, and activated T lymphocytes. In placenta, *MCAM* regulates the migration of intermediate trophoblasts on smooth muscle cells, and in PE placentas, its expression is reduced or undetectable [26]. Here, we provide evidence that the expression of *MCAM* is reduced in the placenta in GDM. However, another study demonstrated an increase in *MCAM* gene expression in GDM women compared to controls. It is worth mentioning that our study excluded women with GDM who required insulin therapy [20], and in the other report, all GDM subjects required chronic insulin therapy for glucose control.

Besides genes involved in cell motility and cell migration, genes involved in the organophosphate biosynthetic process showed significantly altered mRNA expression in the placentas of the GDM women. This process includes the chemical reactions and pathways resulting in the biosynthesis of deoxyribose phosphate. *ADCY5*, *KYNU*, and *TBPL1* were upregulated in the females with GDM compared with the healthy pregnant subjects [27,28,29]. *ADCY5* (adenylate Cyclase 5) is a member of the membrane-bound adenylyl cyclase enzymes that mediate G-protein-coupled receptor signaling through the synthesis of the second messenger cAMP. *ADCY5* is required for glucose coupling to insulin secretion in human islets [27]. Genetic variation within *ADCY5* is associated with impaired insulin secretion, insulin resistance, type 2 diabetes, and GDM [30]. Ustianowski et al. [31] found that the expression of the *ADCY5* gene was significantly increased in the placenta of women with GDM compared with the NGT group. This finding confirms our results.

*KYNU* encodes Kynureninase, a pyridoxal-5’-phosphate dependent enzyme that catalyzes the cleavage of L-kynurenine and L-3-hydroxykynurenine into anthranilic and 3-hydroxyanthranilic acids, respectively. Also, it is involved in the biosynthesis of NAD cofactors from tryptophan through the kynurenine pathway. *KYNU* expression has been identified in endometrium obtained from fertile women and associates with endometrial receptivity to human embryo implantation [28]. To the best of our knowledge, this is the first study describing a higher *KYNU* expression in the placentas of GDM patients compared with NGT women.

*TBPL1* encodes a member of the TATA box-binding protein family. TATA box-binding proteins play a critical role in transcription by RNA polymerase II as components of the transcription factor IID complex. This gene plays a critical role in spermatogenesis, stimulates cell proliferation, and is associated with different cancer types. Additionally, *TBPL1* is closely related to diabetic kidney disease [29]. In our study, for the first time, it was shown that *TBPL1* expression was higher in GDM subjects compared with NGT women. Supportive of this finding is also the different expression of *TBPL1* between normal placenta samples and GDM samples reported by Zhang et al. [32]. These authors used microarray datasets deposited by Binder et al. [33], and after analyzing the Kyoto Encyclopedia of Genes and Genomes (KEGG) pathway enrichment, they found that *TBPL1* was closely related to Herpes simplex infection and to Huntington’s disease.

Finally, this study also showed four DEGs in VAT: the *C4BPA* and *TGOLN2* genes were downregulated and *ACTG2* and *NQO2* were upregulated in the GDM women compared with the NGT subjects [34,35,36]. *C4BPA* (complement Component 4 Binding Protein Alpha) is a multimeric protein that controls activation of the complement cascade through the classical pathway. The complement system plays an important role in inflammation. Several studies have indicated a disorder of complement regulation in GDM [37]. A systematic review and meta-analysis of biomarkers that are differentially expressed in women with and without GDM revealed that *C4BPA* is downregulated in GDM [35]. Similar results were reported in a proteomic analysis of serum exosomes of patients with GDM, in which the authors showed a downregulation of *C4BPA* [34]. Our results support these data.

*TGOLN2* (Trans-Golgi Network Protein 2) may be involved in regulating membrane traffic to and from the trans-Golgi network [38]. High *TGOLN2* expression has been reported in various cancers such as lung adenocarcinoma, squamous cell carcinoma of the maxillary sinus, and esophageal squamous cell carcinoma. Moreover, a variant in the *TGOLN2* gene has been associated with distant metastasis of non-small cell lung cancer [36]. However, to our knowledge, the expression of *TGOLN2* in VAT has not yet been investigated in GDM pregnancies.

*NQO2*, N-Ribosyldihydronicotinamide: Quinone Dehydrogenase 2, is a flavoprotein that catalyzes the two-electron reduction of quinone substrates and uses dihydronicotinamide riboside as a reducing coenzyme [39]. *NQO2* has been shown to be involved in carcinogenesis and neurodegeneration through the regulation of oxidative stress, inflammation, and autophagy [40]. However, the *NQO2* expression in VAT is not known.

*ACTG2* (Actin Gamma 2, Smooth Muscle) is involved in various types of cell motility and in the maintenance of the cytoskeleton. Abnormal *ACTG2* expression was reported in hepatocellular carcinoma and was associated with the aggressiveness of this cancer [41]. Furthermore, a previous study used an animal model to mimic GDM (mice were fed a high-fat diet during pregnancy) and revealed that *ACTG2* was upregulated in the placenta and was strongly associated with other genes [42]. Similarly, the present study showed an upregulation of *ACTG2* in the VAT of women with GDM compared with the NGT participants.

Collectively, our results suggest that transcriptomic alterations in placenta and VAT may play a role in the pathophysiology of GDM. However, it is unclear if the transcriptomic alterations may contribute to GDM or be caused by GDM. Moreover, the findings from this study should be interpreted considering the following limitations. First, there was a relatively low number of participants. Second, all of the patients were Mexican, so the findings might not be applicable to other populations. Third, we did not measure the protein expression levels, and the different cell types in the placenta and VAT were not examined. Further longitudinal studies with a larger sample size and in other populations are required to elucidate the transcriptome profile of GDM. Fourth, although no difference was observed between the GDM and NGT groups with respect to the pre-pregnancy BMI, the BMI values in the NGT group ranged from normal to overweight, whereas the BMI values in the GDM group ranged from overweight to obese. There is increasing evidence that obesity influences gene expression in the blood of individuals with GDM [43]. This influence may also extend to placental gene expression. Finally, our transcriptomic findings related to GDM should be interpreted as those detectable towards the end of pregnancy and persisting despite glycemic control.

## 4. Materials and Methods

### 4.1. Patients and Sample Collection

Between August 2023 and December 2024, five GDM subjects and five normal-glucose-tolerance (NGT) pregnant subjects matched for age, body mass index (BMI) before pregnancy, and gestational age, were recruited from Hospital de Gineco Obstetricia 3, Centro Médico Nacional La Raza, Instituto Mexicano del Seguro Social. The study was conducted in accordance with the Declaration of Helsinki. Signed informed consent was obtained from all patients. The study was approved by the Institutional Review Board from Instituto Mexicano del Seguro Social (R-2023-785-020).

The oral glucose tolerance test (OGTT) with 75 g of glucose was performed in all women between the 24th and 28th week of gestation. GDM patients were diagnosed according to the International Association of the Diabetes and Pregnancy Study Groups criteria, when any one of the following glucose values were met or exceeded: fasting ≥ 5.1 mmol/L, at 1st hour ≥ 10.0 mmol/L and at 2nd hour ≥ 8.5 mmol/L [44]. Eligibility criteria included women with singleton pregnancy and scheduled for an elective cesarean section at term (37–41 weeks of gestation) before the onset of labor. Indications for cesarean section were either breech presentation or previous cesarean section. This study included women with GDM who only received dietary treatment. The exclusion criteria included alcohol or drug abuse, previous diagnosis of type 1 or type 2 diabetes, any form of hypertension, thyroid disease, cardiovascular disease, hepatic or renal disease, and inflammation and infectious diseases. Women with GDM undergoing insulin/metformin treatment were excluded.

Information about maternal age, pre-gestational weight, BMI before and during pregnancy, delivery time, and neonatal data such as sex and birthweight was obtained using medical records. BMI was calculated as the ratio of body weight (kg) to height (m^2^). Both GDM and NGT women were classified as normal-weight (BMI 18.5–24.9 kg/m^2^), overweight (BMI ≥ 25.0 kg/m^2^), or obese (BMI ≥ 30.0 kg/m^2^) according to the criteria of the World Health Organization [45].

Specimens were collected at the time of cesarean section after delivery of the baby within 10 min of operative delivery. Four random samples (1 cm^3^) of the central region of placental tissue on the maternal section and one sample of VAT (1 cm^3^) were collected in RNAlater (ThermoFisher Scientific, Waltham, MA, USA) and were immediately snap-frozen in liquid nitrogen and then stored at −80 °C.

### 4.2. RNA Extraction

Total RNA was isolated from placental tissues and VAT using AllPrep^®^ DNA/RNA/Protein Mini Kit (Qiagen Inc., Hilden, Germany) according to the manufacturer’s instructions. RNA was purified from each of the four areas of the placenta and equitably combined to create a mixture used for gene expression analysis.

### 4.3. RNA Library Sequencing

The RNA integrity of each sample was assessed using an R1 RNA Cartridge for the QSep 400 (BiOptic, New Taipei City, Taiwan). RNA concentration was determined with the Qubit RNA HS Assay Kit (Invitrogen, Carlsbad, CA, USA), while purity was analyzed using a NanoDrop 1000 spectrophotometer (Thermo Fisher Scientific, Wilmington, DE, USA). Transcriptome libraries were prepared with the TruSeq Stranded Total RNA Library Prep with Ribo-Zero Gold (Illumina, San Diego, CA, USA), adjusting fragmentation times based on RIN. The libraries were then quantified using the Qubit dsDNA HS Assay Kit (Invitrogen, Carlsbad, CA, USA), and fragment size was analyzed in the QSep 400 (BiOptic, New Taipei City, Taiwan). Finally, sequencing was performed in a NovaSeq 6000 (Illumina, San Diego, CA, USA) using a 100 bp paired-end configuration.

### 4.4. Read Quality Assessment

Raw reads underwent quality assessment using FastQC 0.12.1. All raw sequences passed the quality control.

### 4.5. Alignment and Gene-Level Quantification of Expression

Reads from 20 libraries (10 of placenta and 10 of VAT) were aligned to the human reference genome (GRCh38) using STAR 2.7.11 [46]. After mapping, gene-level quantification was performed, generating a count table with the HTSeq package 2.0.5 [47]. This count table was used for further differential expression analysis.

### 4.6. Differential Expression and Gene Set Enrichment Analysis

After the placenta libraries were preprocessed (e.g., normalization and filtering), the Principal Component Analysis (PCA) using the first and second components showed that one Control sample behaved anomalously, so we removed it from downstream analysis.

Differential gene expression between groups (GDM vs. NGT) was performed with DESeq2 [48], which is integrated into the iDEP 2.0 online package [49]. DEGs were identified using a threshold of a false discovery rate (FDR) < 0.05 and a fold change > 1.

Finally, the DEGs were subjected to gene set enrichment analysis based on Gene Ontology (GO) databases [50] using all namespaces—molecular function (MF), biological process (BP), and cellular component (CC)—using the gseGO function implemented in the clusterProfiler package 4.12.6 [51].

### 4.7. Validation of Gene Expression Sequencing Data Using qRT-PCR

Ten genes related to cell motility (downregulated mRNAs) and four to the organophosphate biosynthetic process (upregulated mRNAs) were selected for validating the gene expression data in independent placenta samples from 20 NGT mothers and 19 with GDM. One microgram of RNA mixture from placenta was converted to cDNA using SuperScriptTM III First-Strand Synthesis SuperMix kit (ThermoFisher Scientific, Waltham, MA, USA), as recommended by the manufacturer. The qRT-PCR was performed using Taqman^®^ Gene Expression Assays and Taqman^®^ Universal PCR Master Mix on a StepOnePlusTM Real-Time PCR System (ThermoFisher Scientific, Waltham, MA, USA), following the manufacturer’s recommendations. Each reaction was normalized by co-amplification of glyceraldehyde 3-phosphate dehydrogenase (GAPDH). The qRT-PCR data were analyzed using the 2^−ΔCt^ method normalized to GAPDH. All the primers were acquired from ThermoFisher Scientific: *Mmp2* (Hs01548727_m1), *Mmp12* (Hs00159178_m1), *Serpine2* (Hs00299953_m1), *Sema5a* (Hs01549381_m1), *Fn1* (Hs01549976_m1), *Ctgf* (Hs00170014_m1), *Astn2* (Hs01024740_m1), *Mcam* (Hs00174838_m1), *PLXNA4* (Hs00297356_m1), *LYVE1* (Hs00272659_m1), *ADCY5* (Hs02890018_m1), *KYNU* (Hs01114105_m1), *MTMR7* (Hs00952738_m1), *TBP11* (Hs00191595_m1), *GAPDH* (Hs99999905_m1).

### 4.8. Statistical Analyses

Data distribution was assessed using the Shapiro–Wilk test. Differences of characteristics between groups with normal distribution were analyzed using the independent *t*-test and χ^2^ test. The results are presented as mean ± S.D. Data with skewed distribution are presented as the medians with minimum and maximum values and were analyzed using the Mann–Whitney U test. Differences with *p*-values below 0.05 were considered significant. All data were analyzed with the software IBM SPSS Statistics 23.0 (IBM SPSS Inc., Chicago, IL, USA).

## 5. Conclusions

Our study, with the use of sequencing technology, suggests that the placenta plays a crucial role in GDM, as 112 genes were downregulated and 67 genes were upregulated in the placentas of GDM women compared with those of NGT subjects. Genes associated with molecular pathways involved in G-protein-coupled receptor signaling, the organophosphate biosynthetic process, the phospholipid metabolic process, and cell motility and cell migration were altered in the placenta of the GDM women. On the other hand, four DEGs were identified in the VAT. The target pathways of these genes are related to cancer and to the activation of the complement cascade.

## Figures and Tables

**Table 1:** Clinical and biochemical characteristics of the groups studied.

	NGT (n = 5)	GDM (n = 5)	*p*
Age (years)	29.8 ± 6.3	31.0 ± 5.0	0.748
Pre-gestational weight (kg)	68.4 ± 13.0	77.5 ± 8.7	0.236
Pre-pregnancy BMI (kg/m^2^)	28.2 ± 3.4	31.0 ± 3.9	0.254
Pre-pregnancy BMI (n)			
Normal weight	1	0	0.333
Overweight	3	2	
Obesity	1	3	
Current BMI (kg/m^2^)	33.4 ± 4.0	33.2 ± 3.7	0.961
Gestational age at delivery (weeks)	37.9 ± 0.9	38.3 ± 0.9	0.447
Fasting glucose at delivery (mmol/L)	4.45 ± 0.63	4.12 ± 0.43	0.374
Birthweight of newborn (g)	3070 ± 419.2	3330 ± 286.4	0.289
Fetal sex (n)			
Female	1	1	0.778
Male	4	4	

Values represent mean ± SD. NGT, normal glucose tolerance; GDM, gestational diabetes mellitus; BMI, body mass index.

## Data Availability

The original data presented in the study are available in the article and Supplementary Materials. FASTQ files can be provided by contacting the corresponding author.

## Supplementary Materials

The following supporting information can be downloaded at https://www.mdpi.com/article/10.3390/ijms26199595/s1.
